# Green Pea (*Pisum sativum* L.) Hull Polyphenol Extracts Ameliorate DSS-Induced Colitis through Keap1/Nrf2 Pathway and Gut Microbiota Modulation

**DOI:** 10.3390/foods10112765

**Published:** 2021-11-11

**Authors:** Fanghua Guo, Rong Tsao, Chuyao Li, Xiaoya Wang, Hua Zhang, Li Jiang, Yong Sun, Hua Xiong

**Affiliations:** 1 State Key Laboratory of Food Science and Technology, Nanchang University, Nanchang 330047, China; gfh1376234247@outlook.com; 2 Guelph Research and Development Centre, Agricultural and Agri-Food Canada, 93 Stone Road West, Guelph, ON N1G 5C9, Canada; Rong.Cao@canada.ca; 3 Nanchang Inspection and Testing Center, Nanchang 330029, China; ncuspylichuyao@163.com; 4 College of Pharmacy, Jiangxi University of Traditional Chinese Medicine, Nanchang 330004, China; 20211028@jxutcm.edu.cn (X.W.); 20191002@jxutcm.edu.cn (H.Z.); 20152026@jxutcm.edu.cn (L.J.); 5 College of Food Science, Nanchang University, Nanchang 330047, China

**Keywords:** polyphenols, colitis, UHPLC-LTQ-OrbiTrap-MS, GPH extracts, Keap1-Nrf2, gut microbiota

## Abstract

As a processing by-product, green pea hull (GPH) was found to be rich in phenolic components in our previous studies. In this study, UHPLC-LTQ-OrbiTrap-MS (Ultra performance liquid chromatography-linear ion trap orbitrap tandem mass spectrometry) technique was used to quantify polyphenols, and DSS (sodium dextran sulfate)-induced colitis mouse model was established to explore the effect of GPH extracts on colitis. The results showed that quercetin and its derivatives, kaempferol trihexanside and catechin and its derivatives were the main phenolic substances in the extract, reaching 2836.57, 1482.00 and 1339.91 µg quercetin/g GPH extract, respectively; GPH extracts can improved inflammatory status, repaired colonic function, regulated inflammatory factors, and restored oxidative balance in mice. Further, GPH extracts can activate Keap1-Nrf2-ARE signaling pathway, regulate downstream antioxidant protease and gut microbiota by increasing F/B value and promoting the growth of *Lactobacillaceae* and *Lachnospiraceae*, and improve the level of SCFAs (short-chain fatty acids) to relieve DSS-induced colitis in mice. Therefore, GPH may be a promising dietary resource for the treatment of ulcerative colitis.

## 1. Introduction

Ulcerative colitis (UC), a type of inflammatory bowel disease (IBD), is a chronic intestinal inflammation disease with symptoms of abdominal pain, cramps, bloating, diarrhea, constipation and/or blood defecate [1,2]. UC causes a significant decline in the quality of life of patients and is a burden on the medical and health system due to its increasingly higher morbidity, especially in younger patients [1,2,3]. Although several studies have shown that IBD is associated with genetic factors, lifestyle, environment, immune dysfunction, and the intestinal ecosystem, the exact causes have not been definitively confirmed [4,5]. Aminosalicylate, sulfasalazine, mesalazine, glucocorticoids and immunosuppressants are commonly used drugs to treat UC, but long-term use may cause fever, vomiting, acute pancreatitis and other side effects [6,7]. Therefore, new alternative approaches are urgently needed. In fact, diet has been suggested as a treatment for IBD due to the natural active substances in food, such as polyphenols [1,5].

Oxidative stress caused by excessive reactive oxygen species (ROS) produced by activated macrophages and neutrophils is believed to play a vital role in the pathogenesis of colitis [2,8,9]. NF-E2-related factor 2 (Nrf2), a basic leucine zipper transcriptional factor, binds to Kelch-like ECH-associated protein 1 (Keap1) in the cytoplasm under normal conditions, but when activated, it enters the nucleus and binds with the antioxidant responsive element (ARE) to regulate the expression of antioxidant proteins and Phase II detoxification enzymes [2,9,10]. The Keap1-Nrf2-ARE signaling pathway is one of the main cellular defence mechanisms of oxidative stress. Many studies have shown that UC is related to the Keap1-Nrf2-ARE signaling pathway, and activation of Nrf2 may be an alternative strategy for the treatment of colitis [2,9]. Natural polyphenols can act as activators of the Keap1-Nrf2 signaling pathway and inhibit the generation of ROS, increase Nrf2 nuclear translocation and DNA binding capacity, interfere with Keap1-Nrf2 interaction, and promote Keap1 ubiquitination [11]. Therefore, activating the Keap1-Nrf2 signaling pathway through dietary polyphenols may be an effective strategy to prevent or treat UC.

Recently, many studies have shown that intestinal microbial balance is related to UC [2,12,13]. Compared with healthy people, the diversity and abundance of gut microbes in UC patients decreased, which was related to the increased risk of disease recurrence [14]. The disruption of intestinal microecological balance can lead to excessive growth of harmful microorganisms, often manifested in decreased abundance of *Firmicutes* and increased abundance of *Proteobacteria* in colitis [14]. In the colonic tissue of IBD patients, *Bacteroides*, *Lactobacillus,* and *Eubacterium* decreased [15]. Clinically, broad-spectrum antibiotics have been used to treat IBD, but their effects are limited and have side effects [16]. Liu, et al. [17] found that apple polyphenols extract alleviated UC in mice induced with DSS by restoring bile acid metabolism disorder and intestinal flora imbalance. Honey polyphenols [18], chlorogenic acid [19] and caffeic acid [20] can ameliorate mice or rat colitis by regulating the gut microbiota. Ripened Pu-erh tea can promote the production of SCFAs, important gut microbial metabolites, to improve DSS-induced colitis [21]. The existing evidence suggests that a diet rich in bioactive substances can be a promising intervention strategy for the management of UC [13,17,18]. On the other hand, immune dysfunction caused by intestinal mucosal damage was considered one of the leading causes of UC [12,14].

Peas (*Pisum sativum* L.) are widely cultivated in Canada, China, the United States, India, and Russia and are used as a staple food in many areas due to their rich nutrients [22]. Pea hulls, ca. 8−10% of the whole seeds, are a by-product of the industrial production of pea, which is treated as worthless waste [23]. Our previous studies have shown that pea hulls are rich in phenolics and exhibit excellent antioxidant activity against d-galactose-induced oxidative stress in rats [22,23]. So far, we have not seen any research on the effect of pea hull polyphenol extracts on UC. Hence, in the present study, we characterized the main phenolic substances in green pea hull (GPH) extracts by UHPLC-LTQ-OrbiTrap-MS, and assessed the effects of GPH phenolic extracts on UC and gut microbiota based on the Keap1-Nrf2 signaling pathway, using a DSS-induced mouse colitis model. The results of the study may become a potential treatment for UC through an abandoned food resource.

## 2. Materials and Methods

### 2.1. Materials and Reagents

Green pea (ADM Dunn) hulls were provided by Canadian International Grains Institute (CIGI, Winnipeg, MB, Canada) on 21 June 2020. Chromatographic grade methanol, formic acid, and acetonitrile were purchased from Merck (Darmstadt, Germany). Quercetin standard and SCFAs (acetate, propionate, butyrate and valerate) were obtained from Aladdin (Shanghai, China). Dextran sulfate sodium (DSS, molecular weight 36–50 kDa) was purchased from MP Biomedicals (Irvine, CA, USA). The superoxide dismutase (SOD), total antioxidant capacity (T-AOC), catalase (CAT), malondialdehyde (MDA) and myeloperoxidase (MPO) assay kits were purchased from Nanjing Jiancheng Bioengineering Company (Nanjing, China). The enzyme-linked immunosorbent assay (ELISA) kits (TNF-a, IL-6, IL-1β and IL-10) were purchased from Elabscience Biotechnology Co., Ltd. (Wuhan, China). Claudin-1 antibody and Nrf2 antibody was purchased from Affinity (Changzhou, China). ZO-1 antibody and occludin antibody was purchased from Proteintech (Wuhan, China). Keap1 antibody were purchased from GeneTex (Irvine, CA, USA). Primers for glutamate-cysteine ligase catalytic subunit (GCLC), hemeoxygenase-1 (HO-1), and NAD(P)H quinone dehydrogenase 1 (NQO1) were purchased from Tsingke (Beijing, China).

### 2.2. Preparation of GPH Extracts

The GPH extracts were prepared according to our previous method [23]. Briefly, pea hulls were dried by oven at 60 °C and ground into fine powder (through a 200-mesh sieve). The powder was extracted with 80% (*v*/*v*) methanol at a ratio of 1:20 (*w*/*v*) for 30 min at 50 °C by ultrasonication (green apple, China), and then filtered by a vacuum pump. The residue was extracted twice more. The combined filtrate was evaporated at 45 °C by a rotary vacuum evaporator (Eyela N-100, Tokyo, Japan) to ca. 20% of its original volume and then freeze-dried (SIM International Group Co., Ltd., San Jose, CA, USA).

### 2.3. Quantitative Analysis by UHPLC-LTQ-Orbitrap-MS/MS

The liquid chromatographic and mass spectrometric conditions were the same as reported in our previous method with slight modifications [23].

#### 2.3.1. Liquid Chromatographic Conditions

A Thermo Accela 600 UHPLC system (Thermo Scientific, Bremen, Germany) equipped with a binary pump and an autosampler was used. Separation was performed on a C18 column (2.1 × 150 mm, 2.8 μm particle size; ACCHROM) operated at room temperature. The binary mobile phase consisted of A (water containing 0.1% formic acid, *v*/*v*) and B (acetonitrile containing 0.1% formic acid, *v*/*v*). The flow rate was 0.30 mL/min, and the injection volume was 10 μL. The gradient elution program was as follows: 0−2 min, 5% B; 2−14 min, 5−33% B; 14−18 min, 33−48% B; 18−25 min, 48−100% B; 25−27 min, 100−100% B; 27−31 min, 100−5% B.

#### 2.3.2. Mass Spectrometric Conditions

Mass spectrometry data were obtained using an LTQ-OrbiTrap-MS equipped with a heated-electrospray ionization probe (HESI-II; Thermo Fisher Scientific, Waltham, MA, USA) operated in the negative mode. All compounds are tentatively identified based on congruent retention times (t_R_), accurate molecular mass, predicted molecular formula, fragmentation pattern with data in the literature, databases. Parameters of the ion source were set as follows: source voltage 5 kV, capillary voltage −40 V, tube lens voltage −80 V, capillary temperature 275 °C, sheath, and auxiliary gas flow (N2) was at 42 and 11 (arbitrary units). Mass spectra from 100 to 2000 *m*/*z* were acquired. Collision induced dissociation (CID) experiments were conducted for the fragmentation study. The normalized collision energy of the CID was set at 45 eV. Accurate mass analysis was calibrated according to the manufacturer’s guidelines.

#### 2.3.3. Calibration and Quantification

The quantification of the main phenolic compounds in GPH extracts was based on the peak area of quercetin under the same conditions of mass spectrometry, which was a semi-quantitative method and expressed as quercetin equivalents (μg quercetin/g GPH extract). Briefly, the accurately weighed quercetin was dissolved in 80% methanol (20% water, *v*/*v*) and diluted to a certain concentration, and a standard curve was prepared based on the peak area. Sample peak area was integrated according to molecular weight and retention time.

### 2.4. Experimental Animals

Twenty-six male C57BL/6 mice (7 weeks old; 18–20 g), purchased from Liaoning Changsheng Biotechnology Co., Ltd. (Changchun, China, SCXK 2020-0001), were raised in an air-conditioned room (23 ± 2 °C, 12 h light/dark cycle) for 7 days with free access to food and water before the experiments. The mice were divided into four groups as follows: 

Group I (control, *n* = 6): the control group (treated with normal saline);

Group II (DSS, *n* = 8): the model group (treated with normal saline);

Group III (LGPH, *n* = 6): the LGPH group [treated with 100 mg extracts per kg body weight (100 mg/kg BW/d)];

Group IV (HGPH, *n* = 6): the HGPH group [treated with 600 mg extracts per kg body weight (600 mg/kg BW/d)].

During the experiment, all mice ate a normal diet and drank water containing 3% DSS (except for Group I) to induce colitis from the eighth day. The schematic diagram of the experiment was shown in Figure 2A. The body weight, feces status, bloody stool, and activity states (vitality, coat condition, posture, behavior) of mice were observed and recorded daily. On the 14th day, the mice were fasted for 4 h after drinking 3% DSS and were sacrificed by cervical dislocation. Colon tissue, colon contents and feces were collected for analysis. All animal experimental procedures were approved by the Animal Ethics Committee of Nanchang University.

#### 2.4.1. Disease Activity Index (DAI) Assessment

DAI was a summation of weight loss, stool hardness, rectal bleeding, and activity status scores and was scored from the eighth day. The scoring standards were adapted from a previous study [24], as shown in Table S1.

#### 2.4.2. Evaluation of Histological Changes

The colon was quickly taken out from the sacrificed mice, washed with saline, and measured for length. The colon tissues dehydrated by gradient alcohol were fixed with paraffin, sliced, and stained with hematoxylin and eosin (H&E). Colonic tissues were evaluated under light microscopy according to scoring criteria shown in Table S2 [25].

#### 2.4.3. MPO Assessment

The MPO activity, a key indicator of neutrophil infiltration, in the colon was assessed by a MPO Test Kit according to the manufacturer’s instructions. The colon tissues were washed with pre-cooled PBS (0.01 M, pH = 7.4), weighed, and ground in an ice bath. The homogenate was centrifuged for 10 min at 5000 rpm and the supernatant was detected at 460 nm for absorbance. Experiments were carried out three times, and the results were expressed as units per gram of tissue (U/g tissue).

#### 2.4.4. Oxidative Stress Markers

The protein concentration of the supernatant from colon tissues were determined with the bicinchoninic acid (BCA) kit (Beyotime Biotechnology, Shanghai, China). SOD, T-AOC, CAT and MDA of colon tissue were assessed with commercial kits.

#### 2.4.5. Inflammatory Cytokines

The levels of inflammatory mediators (TNF-a, IL-6, IL-1β, and IL-10) of the colon tissues were measured by ELISA kits according to the manufacturer’s instructions.

#### 2.4.6. Western Blot Analysis

The protein expression levels of ZO-1, claudin-1, occludin, GAPDH, Keap-1, Nrf2 (cytoplasm and nucleus) and lamin-B were measured by Western blot analysis. Protein extraction from colon tissue was performed according to the kit instructions (P0027, Beyotime Biotechnology, Shanghai, China) and the concentration was determined with the BCA kit (Solarbio Science and Technology Co., Ltd., Beijing, China). The protein was separated by SDS-PAGE gel and then transferred to the PVDF membrane, which was cut into different bands according to the molecular weight of the protein. The bands were blocked by TBST containing 5% skimmed milk powder for 2 h, and then diluted primary antibody was added and incubated overnight at 4 °C. The excess primary antibody was washed off with TBST and the secondary antibody was incubated at room temperature for 2 h. Protein bands were visualized using the enhanced chemiluminescence solution and grey value was analyzed.

#### 2.4.7. Quantitative Real-Time Polymerase Chain Reaction (PCR) Analysis

Total RNA was extracted from the colon tissues by TRIzol reagent (Thermo Scientific, Waltham, MA, USA) according to the manufacturer’s instructions. The primer sequences are shown in Table 1. The cDNA was obtained by reverse transcription of total RNA at 25 °C for 5 min, 50 °C for 15 min, 85 °C for 5 min, and 4 °C for 10 min with PCR gene amplification instrument (EDC-810, Dongsheng, China). Real-time PCR was performed with the Applied Biosystems^®^ QuantStudio™ 6 Flex Real-Time PCR System. Relative mRNA expression levels of GCLC, HO-1 and NQO1 were normalized with GAPDH and calculated with the 2^−^^ΔΔCt^ method.

#### 2.4.8. SCFAs Analysis

A DB-1701 chromatographic column (30 m × 0.25 mm × 0.25 μm, Analytical Technology, Chromatography Technology Research and Development Center, Lanzhou Institute of Physical Chemistry, Chinese Academy of Sciences, Lanzhou, China), was used to determine the content of SCFAs in feces. Briefly, a fecal sample (0.1 g) was mixed with 1 mL water, followed by ultrasound for 10 min (100 W), standing for 20 min and centrifugation for 15 min (10,000× *g*, 4 °C). The precipitate was mixed with 0.5 mL water and extracted again. The combined supernatants were then filtered through 0.22 μm membrane, and injected (2 μL) automatically into the inlet at a temperature of 240 °C with a split ratio of 8:1. The temperature program was as follows: the initial temperature was 80 °C and maintained for 0.5 min, then increased to 140 °C at a rate of 30 °C/min and maintained for 0.5 min; then increased to 190 °C at a rate of 5 °C/min and maintained for 0.5 min; then increased to 230 °C at a rate of 60 °C/min and maintained for 1 min. The content of SCFAs in feces was quantified based on the standard curves.

#### 2.4.9. Gut Microbiota Analysis

For 16S rDNA gene sequencing, the fecal samples from colon were sent to Yuewei Gene Technology Co., Ltd. (Beijing, China). After total bacterial genomic DNA was extracted, the V3-V4 region of bacterial 16S rRNA was amplified by PCR, and the PCR products were purified using QIAGEN Gum Recovery Kit (Qiagen, Hilden, Germany). High-quality clean reads were sequenced with HiSeq2500-PE250 (Illumina, CA, USA). Sequencing data were analyzed based on Greengenes (http://greengenes.lbl.gov/, accessed on 23 February 2021) and Quantitative Insights into Microbial Ecology (QIIME, V1.9.1).

### 2.5. Statistical Analysis

Data were expressed as the mean ± standard deviation and the level of *p* < 0.05, calculated by one-way analysis of variance (ANOVA) followed by Dunnett’s test with SPSS (version 25.0, IBM, Armonk, NY, USA), was considered statistically significant. The pictures were drawn by Origin software (version 2019b, USA).

## 3. Results

### 3.1. Quantification of Polyphenols in GPH Extracts

**Figure 1:**
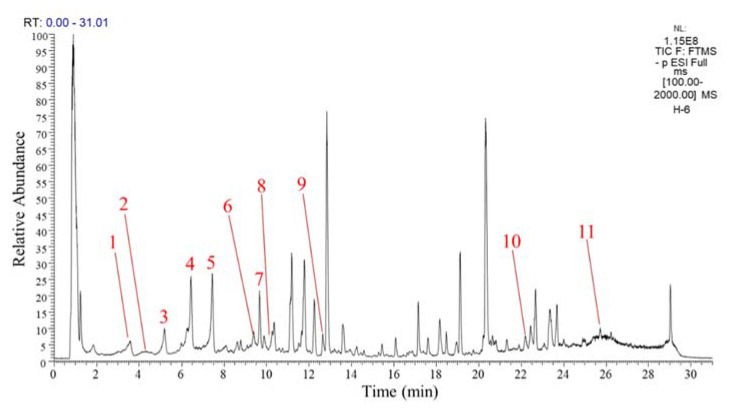
Total ion chromatograms (TIC) of GPH extracts.

Our previous study showed that GPH extracts mainly contained flavonoids and their derivatives and could alleviate oxidative stress induced by d-galactose in rats [23]. Therefore, the main phenolic substances in the GPH extracts were quantified using the UHPLC-LTQ-Orbitrap-MS/MS method. The content of flavonoids and their derivatives were quantified as quercetin equivalent, and the calibration curve was y = 30,000,000x + 276,499 (R^2^ = 1.000). A total of 11 substances were quantified, as shown in Figure 1 and Table 2. Peaks 1, 2, and 3 were tentatively identified as (Epi)catechin conjugate 1, (Epi)gallocatechin dimer, and (Epi)catechin conjugate 2 with contents of 496.53 ± 30.60, 214.68 ± 15.01 and 628.70 ± 40.06 µg quercetin/g GPH extract, respectively. Peaks 4 and 5 containing 1409.24 ± 60.01 and 1381.57 ± 91.18 µg quercetin/g GPH extract were tentatively identified as quercetin derivative. Peaks 6 and 7 were two different kaempferol trihexoside with contents of 492.17 ± 36.28 and 989.83 ± 53.77 µg quercetin/g GPH extract. Peaks 10 and 11 were tentatively identified as isorhamnetin glycoside and naringenin with contents of 294.18 ± 25.20 and 239.15 ± 13.00 µg quercetin/g GPH extract. Small amounts of quercetin 3-glucoside (peak 8, 45.76 ± 5.02 µg quercetin/g GPH extract) and theaflavin derivative (peak 9, 23.34 ± 2.52 µg quercetin/g GPH extract) were quantified in GPH extracts. Quercetin and its derivatives had the highest contents (2836.57 µg quercetin/g GPH extract), followed by kaempferol trihexoside (1482.00 µg quercetin/g GPH extract) and catechin and its derivatives (1339.91 µg quercetin/g GPH extract).

### 3.2. Anti-Inflammatory Effects of GPH Extracts in Mice with DSS-Induced Colitis

#### 3.2.1. GPH Extracts Mitigated the Symptoms of DSS-Induced UC Mice

**Figure 2:**
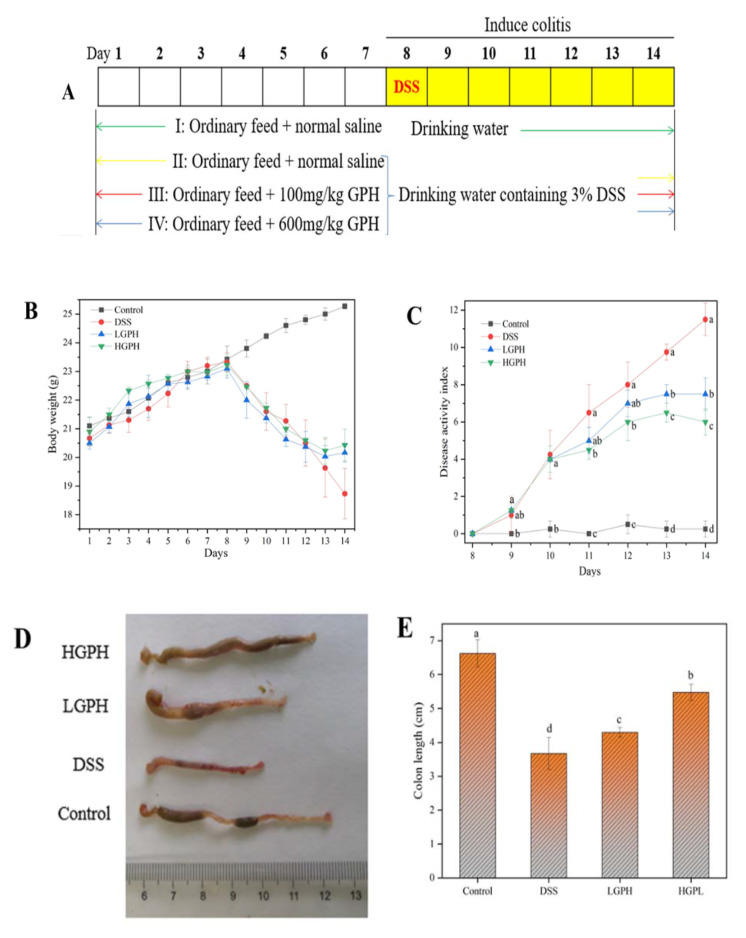

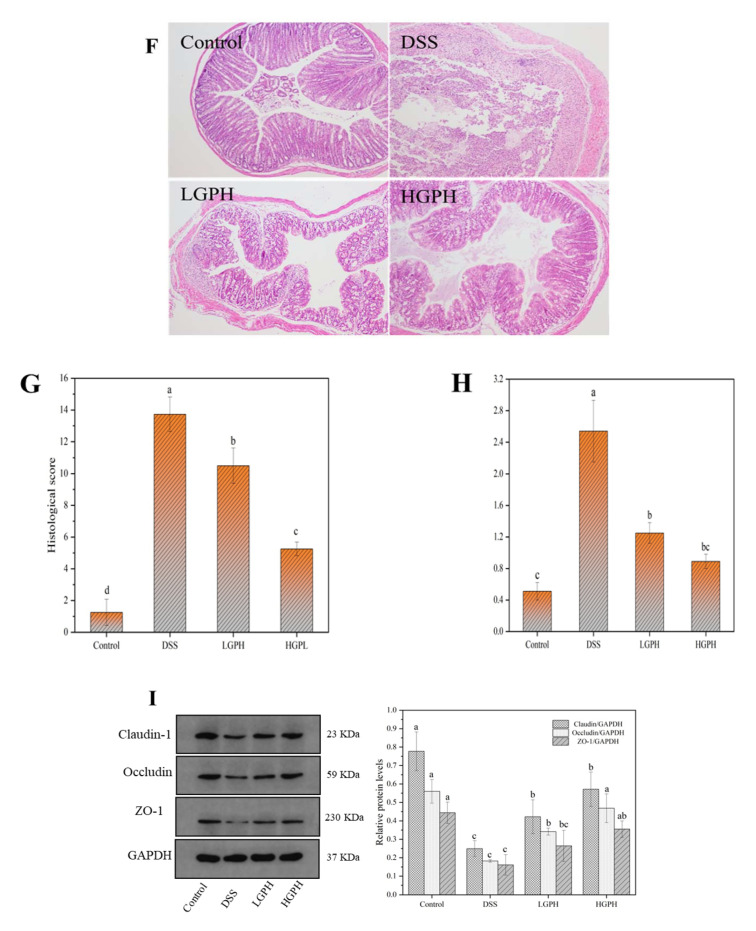
GPH extracts mitigated the symptoms and relieved the colonic injury and inflammatory infiltration of DSS-induced UC mice. (**A**) Schematic diagram of the experiment; (**B**) body weight; (**C**) DAI score; (**D**) representative photographs of colon length; (**E**) quantitative results of colon length; (**F**) representative photographs of H&E staining of colonic tissues (magnification ×100); (**G**) histopathological score of colonic tissues; (**H**) MPO activity of colonic tissues; (**I**) expression of tight junction proteins in colonic tissues. Values not sharing a common superscript letter denote significant difference (*p* < 0.05).

Under the continuous induction by 3% DSS, the mice developed obvious colitis symptoms, such as diarrhea, blood in the stool, weight loss. As shown in Figure 2B, the weight of the mice dropped sharply except for the control group from the 8th day; but from the 13th day, the weight of the LGPH and HGPH groups began to recover compared with the DSS group. DAI is an essential indicator of clinical symptoms of colitis. From Day 10, DAI scores in the DSS, LGPH and HGPH groups were significantly higher than those in the control group, while colitis began to recover in the LGPH and HGPH groups on Day 12 (Figure 2C, *p* < 0.05). The colons of the DSS and LGPH groups were bright red, which indicates that the mice have blood in the stool (Figure 2D). Colon length was significantly shortened in the DSS group (3.68 ± 0.47) compared with the control group (6.63 ± 0.40), but the length was restored after treatment with GPH extracts (LGPH: 4.30 ± 0.14; HGPH: 5.48 ± 0.24) and the effect of high concentration was more significant (Figure 2E, *p* < 0.05). Data of body weight, DAI, stool blood and colon length showed that GPH extracts improved colitis in mice, and the high concentration of GPH extracts was better than low concentration.

#### 3.2.2. GPH Extracts Relieved the Colonic Injury and Inflammatory Infiltration in DSS-Induced UC Mice

H&E staining, MPO and tight junctions (TJ) were used to evaluate the effects of GPH extracts on colonic tissue of ulcerative mice. Mice of the DSS control had their colonic tissues severely damaged, showing lymphocyte infiltration, epithelial cell shedding, crypt loss, and mucosal ulceration, whereas none of these symptoms was seen in the normal control group (Figure 2F). However, the aforementioned colonic tissue damages of mice treated with a high dose of GPH extract were significantly ameliorated, which was further evidenced in significantly lowered histopathological score (Figure 2G, *p* < 0.05). 

A pro-oxidative and pro-inflammatory enzyme, MPO is mainly secreted by activated neutrophils [26]. As shown in Figure 2H, MPO activity in colonic tissues increased nearly five-fold after exposure to DSS (2.54 ± 0.39); however, it was a significantly reduced after GPH extracts treatment, both at low (1.25 ± 0.13) and high doses (0.89 ± 0.09, *p* < 0.05). 

The integrity of intestinal epithelium is of great significance to the prevention of IBD [27]. Tight junctions facilitate the epithelial cells to form the epithelial layer that functions an important physical intestinal barrier [27]. As shown in Figure 2I, DSS treatment significantly down-regulated the expression of claudin-1, occludin and ZO-1 proteins compared with the control group (*p* < 0.05). By contrast, except for ZO-1 at low concentrations, the expressions of the three tight junction proteins were significantly increased after treatment with low-dose and high-dose GPH extracts (*p* < 0.05). Our results indicate that treatment with GPH extracts might show appreciably beneficial effects in reducing DSS-induced intestinal damage.

#### 3.2.3. GPH Extracts Protected Colon from Oxidative Stress in DSS-Induced UC Mice

**Figure 3:**
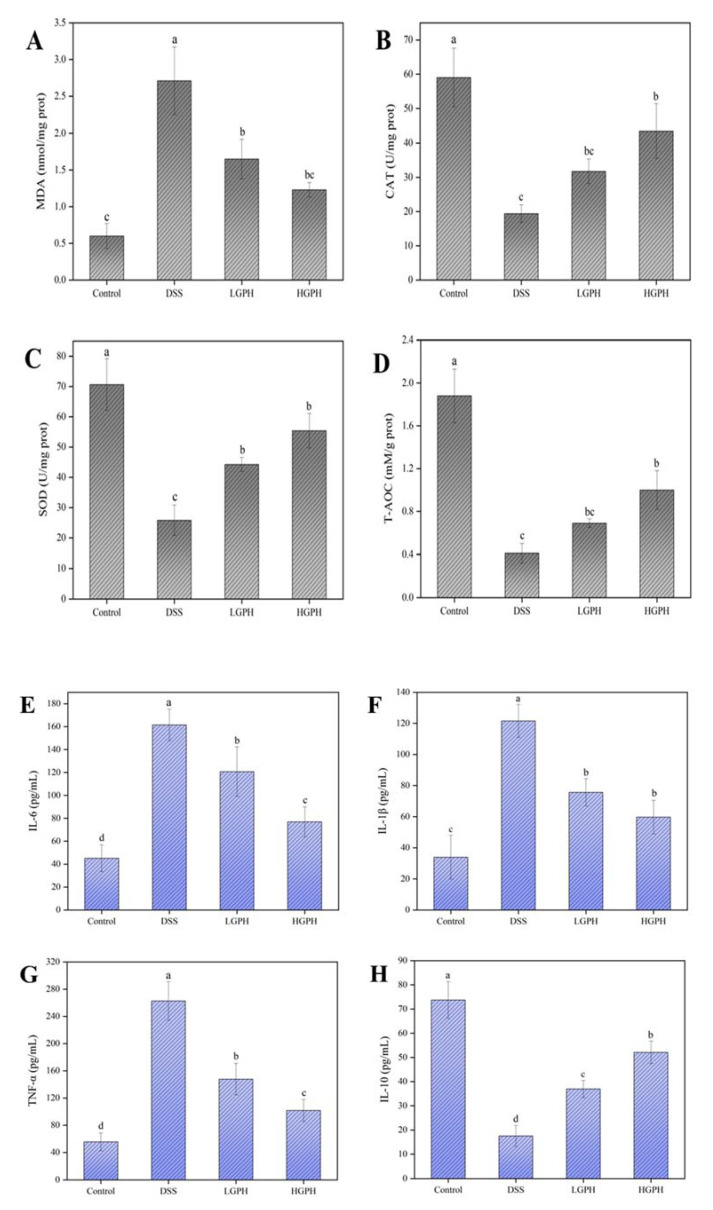
GPH extracts protected colon from oxidative stress and improved the inflammatory status in DSS-induced UC mice. (**A**) MDA; (**B**) CAT; (**C**) SOD; (**D**) T-AOC; (**E**) IL-6; (**F**) IL-1β; (**G**) TNF-α; (**H**) IL-10. Values not sharing a common superscript letter denote significant difference (*p* < 0.05).

To evaluate the effect of GPH extracts on oxidative stress in DSS-induced colitis in mice, MDA, CAT, SOD and T-AOC were measured. As shown in Figure 3A–D, with DSS treatment, MDA content increased significantly, and SOD, CAT and T-AOC decreased significantly compared with the normal control group (*p* < 0.05), which indicates that DSS has effectively induced oxidative stress in the colon. However, mice both LGPH and HGPH groups showed significantly reduced MDA content and enhanced the activity of SOD (Figure 3A,C, *p* < 0.05); high-dose GPH extracts treatment also significantly increased CAT and T-AOC activities (Figure 3B,D, *p* < 0.05) compared with the DSS group, suggesting that GPH extracts are beneficial to restore colonic oxidative balance.

#### 3.2.4. GPH Extracts Improved the Inflammatory Status in DSS-Induced UC Mice

TNF-α, IL-1β, IL-6, and IL-10 were tested with ELISA kits to clarify the effect of GPH extracts on cytokines in the colon of ulcerated mice. The content of pro-inflammatory factors (TNF-α, IL-1β, and IL-6) increased significantly under DSS induction; however, after treatment with GPH extracts, they were reduced markedly and the effect was concentration-dependent (Figure 3E–G, *p* < 0.05). As shown in Figure 3H, IL-10 levels decreased from 73.71 ± 7.60 pg/mL (control group) to 17.54 ± 4.31 pg/mL (DSS group), but the DSS-induced decline in IL-10 was significantly recuperated after intervention with low and high doses of GPH extracts, to 36.95 ± 3.50 pg/mL (LGPH group) and 52.08 ± 4.67 pg/mL (HGPH group), respectively.

#### 3.2.5. GPH Extracts Activated the Keap1/Nrf2 Signaling Pathway and Promoted the mRNA Expression of Nrf2 Downstream Genes

**Figure 4:**
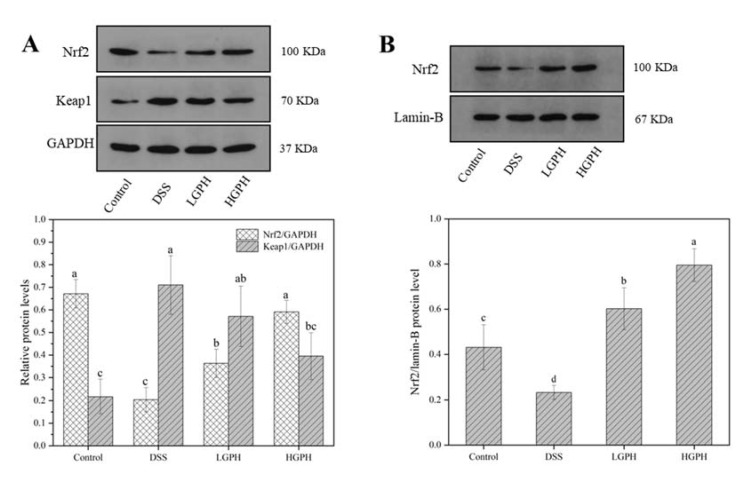

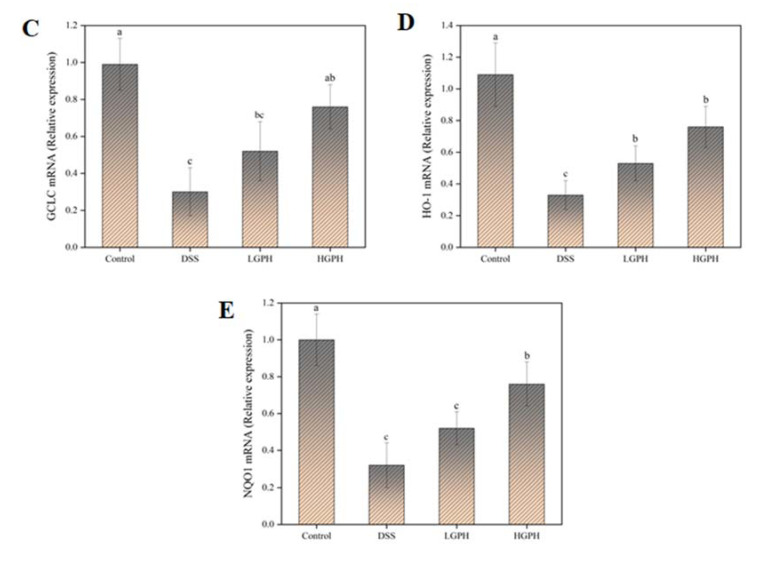
GPH extracts activated the Keap1/Nrf2 signaling pathway and promoted the mRNA expression of Nrf2 downstream genes. Relative protein expression levels of (**A**) Keap1 and cytosolic Nrf2; (**B**) Nuclear Nrf2. Relative mRNA expression levels of (**C**) GCLC; (**D**) HO-1; (**E**) NQO1. Values not sharing a common superscript letter denote significant difference (*p* < 0.05).

To investigate whether GPH extracts alleviate colitis by regulating the Keap1/Nrf2 signaling pathway, the protein expression levels of Keap1 and Nrf2 (cytoplasmic and nuclear) in colonic tissues were analyzed using the Western blot. As shown in Figure 4A, compared with the control group, the cytoplasmic Keap1 protein expression level in the DSS group was significantly increased (*p* < 0.05), but GPH extracts reduced the DSS-induced increase of the cytoplasmic Keap1 protein expression, particularly at the higher dose (Figure 4A). The expressions of Nrf2 protein in cytoplasm and nucleus were decreased with DSS treatment but were significantly increased by the GPH extracts treatment in both LGPH and HGPH groups (Figure 4A,B), suggesting that the polyphenols of the GPH extracts may combine with the Keap1 to promote the nuclear transfer of Nrf2 [28].

When Nrf2 is transferred to the nucleus, it will promote the expression of downstream antioxidant-related proteins. GCLC, HO-1 and NQO1 gene expression levels were significantly reduced by the DSS treatment (*p* < 0.05), but they were significantly recovered by the high-dose GPH extracts treatment compared with the DSS group (Figure 4C–E, *p* < 0.05), and these results were consistent with the changes of Nrf2 in the cytoplasm.

#### 3.2.6. GPH Extracts Increased SCFAs Content in DSS-Induced UC Mice

**Figure 5:**
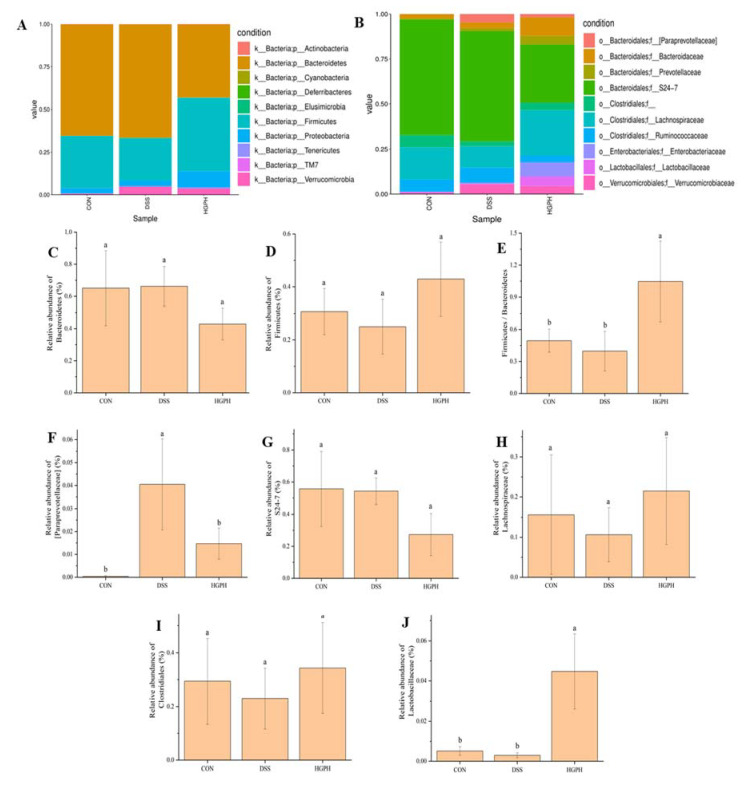

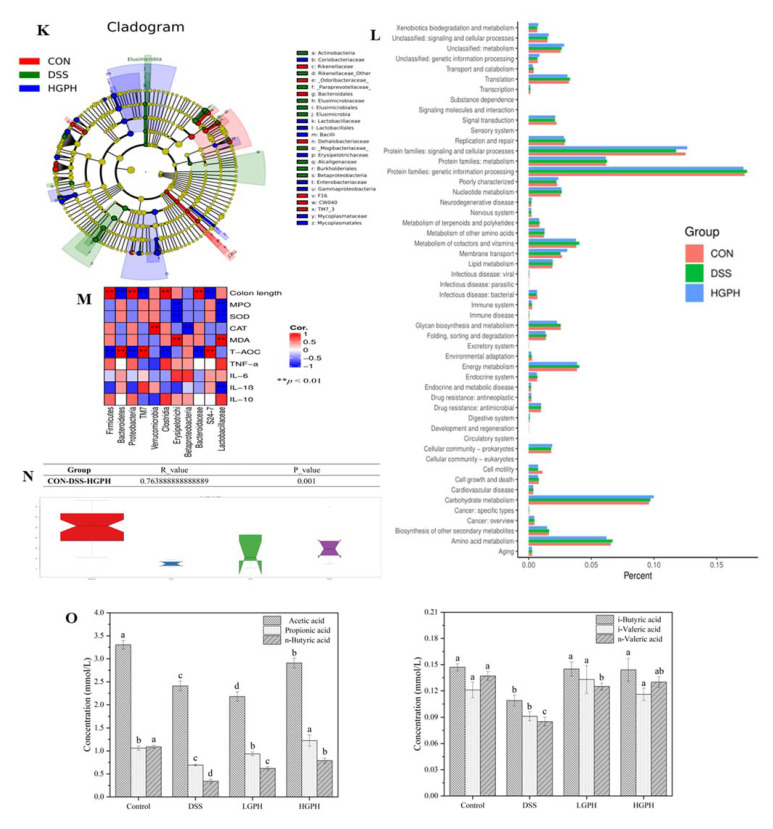
GPH extracts altered gut microbiota in mice with DSS-induced colitis. Gut microbiota composition at the phylum level (**A**) and family level (**B**); different groups of differential microbial taxa at the phylum level (**C**,**D**) and family level (**F**–**J**); the ratio of Firmicutes/Bacteroidetes (F/B) at the phylum level (**E**); taxonomic cladogram obtained from LEfSe analysis of 16S sequences (**K**); KEGG pathway analysis (**L**); Spearman’s rank correlation coefficients (**M**); ANOSIM analysis (**N**); SCFAs content (**O**). Values not sharing a common superscript letter denote significant difference (*p* < 0.05).

As shown in Figure 5O, the SCFAs in mouse feces were mainly acetic acid, propionic acid, and *n*-butyric acid. In contrast, the contents of *i*-butyric acid, *n*-valeric acid and *i*-valeric acid were low. Under DSS treatment, the contents of the six SCFAs were significantly reduced compared with the control group (*p* < 0.05). After the intervention of low dose GPH extracts, the contents of SCFAs were recovered significantly, except for acetic acid, while the high-dose can increase the content of all six SCFAs (*p* < 0.05). 

### 3.3. GPH Extracts Altered Gut Microbiota in Mice with DSS-Induced Colitis

The effect of GPH extracts on the gut microbiota in mice with DSS-induced colitis was analyzed with the 16S rDNA sequencing technology. At the phylum level (Figure 5A), *Bacteroidetes*, *Firmicutes*, *Proteobacteria* and *Verrucomicrobia* were the four main types of gut microbiota, accounting for about 99% of the total abundance in control (CON), DSS and HGPH groups. *Bacteroidetes* and *Firmicutes* were two important dominant intestinal bacteria, and their abundance changed in the opposite direction, i.e., DSS treatment increased the *Bacteroidetes* (0.66 ± 0.12) but decreased the *Firmicutes* (0.25 ± 0.10) population, whereas the opposite was true for effect by high concentration GPH extracts (0.43 ± 0.10 and 0.43 ± 0.14, respectively), compared with the control group (0.65 ± 0.23 and 0.31 ± 0.09, respectively; Figure 5C,D). Intervention with high concentration of GPH extracts also significantly restored the ratio of *Firmicutes*/*Bacteroidetes* (F/B, 1.05 ± 0.38) (Figure 5E, *p* < 0.05). At the family level, the 10 major intestinal bacteria are shown in Figure 5B. Compared with the CON group, the relative abundance of *Paraprevotellaceae* was increased by DSS (0.040 ± 0.020) but there was an obvious decrease in the HGPH group (0.015 ± 0.0068) (Figure 5F). As a probiotic, the abundance of *Lactobacillaceae* was inhibited by DSS (0.0029 ± 0.0013), but the abundance was significantly increased following treatment with HGPH treatment (0.045 ± 0.019) (Figure 5J, *p* < 0.05). As shown in Figure 5H,I, DSS reduced the abundance of *Lachnospiraceae* (0.11 ± 0.068) and *Clostridiales* (0.23 ± 0.11), which were associated with the production of SCFAs, but their abundance was restored after treatment with HGPH extracts (0.21 ± 0.13 and 0.34 ± 0.17, respectively). Interestingly, the abundance of *S24-7* in the HGPH group (0.27 ± 0.13) was lower than that in the DSS (0.54 ± 0.082) and CON (0.56 ± 0.23) groups (Figure 5G).

To further analyze the relationship between the microbiota of mice in each group and UC, linear discriminant analysis effect size (LEfSe) analysis was used across from phylum to genus (Figure 5K). *Paraprevotellaceae*, *Elusimicrobia* and *Betaproteobacteria* were identified as the dominant intestinal flora in the DSS group. *Rikenellaceae*, *Bacteroidales* and *Odoribacteraceae* were the pivotal phylotypes of gut microbiota in the CON group. In addition, compared with other groups, *Bacilli*, *Lactobacillaceae* and *Gammaproteobacteria* were considered the enriched bacterial taxa. The results of ANOSIM were shown in Figure 5N, which indicated a significant change of gut microbial structure among the three groups (R = 0.76 > 0, *p* < 0.05). The KEGG pathways were analyzed with the phylogenetic investigation of communities by reconstruction of unobserved states in three groups. As shown in Figure 5L, the main functional profiles affected were genetic information processing, signaling and cellular processes, carbohydrate metabolism, amino acid metabolism, energy metabolism, metabolism of cofactors and vitamins, translation, replication and repair, membrane transport, and signal transduction, all of which belonged to metabolism and information processing.

A correlation analysis between the altered intestinal bacteria, inflammatory cytokines and oxidative stress markers was performed with Spearman’s rank correlation coefficient. All 11 gut microbes were positively or negatively correlated with at least one inflammatory factor or oxidative stress marker (Figure 5M). Colon length was positively correlated with *Firmicutes*, *Proteobacteria*, *Clostridia*, and *Betaproteobacteria*, while negatively correlated with *Bacteroidetes*, *TM7*, and *S24-7*, which was contrary to T-AOC. *Erysipelotrichi* and *Lactobacillaceae* were negatively correlated with MPO and SOD, while positively correlated with MDA. *Clostridia* and *Lactobacillaceae* were positively correlated with IL-10, while *Erysipelotrichi* and *Betaproteobacteria* were positively correlated with IL-6.

## 4. Discussion

As a processing by-product, GPH was found to be rich in polyphenols in our previous studies, containing mainly catechin, kaempferol, quercetin and its derivatives, but there was no quantitative study on it [23]. UHPLC-LTQ-Orbitrap-MS method is an efficient and rapid modern analysis technology established in recent years. It can accurately conduct qualitative and quantitative research on a variety of complex systems because of its ability to collect multistage mass spectral information [23]. In this study, the main phenolic compounds in the GPH extracts were quantified (quercetin as the standard) using UHPLC-LTQ-Orbitrap-MS method. These main polyphenols, including quercetin and its derivatives, kaempferol trihexoside and catechin and its derivatives, are known to have excellent antioxidant and anti-inflammatory activities in vitro and in vivo [22,23]. A study has found that *T. articulata*, a plant rich in catechin, has potent antioxidant, anti-inflammatory and antibacterial activities, which makes it an interesting matrix in the development of novel pharmaceutical formulations [29]. Lesjak et al. [30] studied different quercetin and its derivatives and found that they all had good antioxidant and anti-inflammatory activities, and they were present in the systemic circulation, which suggested that bioavailability and its metabolites should be taken into account when defining the activity of active substances. Our previous study showed that GPH extracts could alleviate oxidative stress induced by d-galactose in rats, and 49 kinds of GPH extract metabolites were found in plasma and urine [23]; on the other hand, there were 8, 23, 8, and 20 yellow pea hull (YPH, Canada) extract metabolites detected, mainly belonged to kaempferol, quercetin, and catechin, in heart, liver, lung and kidney tissues, respectively, and these tissues exhibited good antioxidant activity (SOD, GSH-Px, MDA and T-AOC) [22]. Therefore, we believe that GPH extract reduces DSS-induced colitis in mice due to the combined effect of the intrinsic polyphenols and their metabolites.

DSS is often used to establish mouse colitis models, which have similar symptoms to patients with colitis, including weight loss, diarrhea, and blood in the stool [31]. DAI score and colon length are important indicators of the severity of colitis [24,25]. In this study, treatment with GPH extract alleviated DSS induced colitis in mice, manifested as weight recovery, increased colon length, decreased DAI score, and a dose-dependent effect (Figure 2). In the pathogenesis of colitis, inflammation was exacerbated by the disruption of the intestinal epithelial barrier, often shown in reduction of mucus and goblet cells, and damage to the colonic mucosa [31,32]. There were severe lymphocyte infiltrations, epithelial cell shedding, crypt loss and mucosal ulcers in the colon tissue of inflammatory mice; however, these symptoms were improved after GPH extracts treatment and histological score was significantly reduced (*p* < 0.05). On the other hand, MPO activity is an important indicator reflecting the infiltration of neutrophils into colon tissue. In our current research, GPH extracts can significantly reduce the level of MPO in colon tissue, especially high-dose GPH extracts (*p* < 0.05). From these apparent indicators, GPH extracts treatment can alleviate colitis in mice and show a significant protective effect.

Intestinal epithelial cells connected by TJ proteins were a key barrier to prevent pathogenic microorganisms, pathogenic antigens, and toxic substances from entering the systemic circulation from the intestinal lumen [33]. Maintaining the normal expression of TJ proteins and the permeability of the intestinal epithelial barrier may be a crucial target for the treatment of IBD. Consistent with the previous results, the protein expression of claudin-1, occludin, and ZO-1 decreased after treatment with DSS [34]. However, treatment with the GPH extracts significantly increased the expression of the three TJ proteins, possibly due to the richness of flavonoids in the extracts. Many studies have shown that dietary polyphenols protect TJ proteins. Chen and Kitts found that orange peel extracts rich in flavonoids can improve tight junction dysfunction induced by alcohol [35]. As the most abundant flavonoid, quercetin can enhance epithelial barrier function and increase claudin-4 expression in Caco-2 cells [36]. Therefore, ingestion of GPH extract can restore the intestinal barrier function in colitis mice.

A large number of studies have shown that IBD is related to the imbalance of intestinal immunity and inflammatory factors, such as IL-6, IL-1β and TNF-α, which are involved in the destruction of TJ proteins and are regulated by the NF-kB signaling pathway [2,31,34]. Therefore, controlling the production and release of inflammatory cytokines is considered a potential strategy for the treatment of IBD. In this study, consistent with IBD patients, high levels of pro-inflammatory factors (IL-6, IL-1β and TNF-α) were found in mice with DSS-induced colitis, while these pro-inflammatory factors were reduced after treatment with GPH extracts, and high-dose extracts had a stronger inhibitory effect. Simultaneously, the level of IL-10 inhibited by DSS increased significantly after the intervention of GPH extracts (*p* < 0.05). The results indicated that the ameliorative effect of GPH extracts on DSS-induced colitis in mice may be related to the maintenance of inflammatory cytokine balance.

Oxidative stress, caused by the imbalance between antioxidants and pro-oxidants, is one of the important causes of colitis [2]. Antioxidant enzymes, such as CAT and SOD, are important defense systems in the body. The levels of CAT and SOD reduced by DSS were significantly increased after treatment with GPH extract (*p* < 0.05). Under continuous DSS exposure, the content of MDA in colon tissue was significantly increased compared with the control group, indicating that lipid peroxidation was induced (*p* < 0.05). GPH extract might relieve colonic oxidative stress by reducing MDA and increasing T-AOC content, which was consistent with our previous research [23].

The Keap1-Nrf2-ARE signaling pathway is one of the most important defense systems against oxidative and/or electrophilic stress [11]. An increasing number of recent studies have shown that the Keap1-Nrf2-ARE signaling pathway is closely related to colitis [2,4,9]. Under normal physiological conditions, Keap1 binds to Nrf2 and is ubiquitinated in the cytoplasm. Under stress conditions, the Keap1 protein is covalently modified by excessive oxidation and/or electrophiles, resulting in conformational changes, which inhibits the ubiquitination of Nrf2 [9]. Subsequently, Nrf2 enters the nucleus and binds to ARE, activating the expression of downstream antioxidant proteins. DSS inhibited Nrf2 levels and increased Keap1 levels; however, treatment with GPH extracts reversed the results. In the nucleus, in both low-dose and high-dose GPH extracts treatment groups, the levels of Nrf2 were significantly increased compared with the DSS group, which was consistent with the downstream antioxidant protein gene expression levels (GCLC, HO-1 and NQO1) (*p* < 0.05). Therefore, GPH extracts may restore oxidative balance and alleviate colitis by activating the Keap1/Nrf2 signaling pathway, which may be related to the rich polyphenols in the extracts. Studies have shown that polyphenols can not only inhibit ROS, but also degrade Keap1, inhibit the combination of Keap1 and Nrf2, and activate the Keap1-Nrf2 signaling pathway [11]. Sun et al. [28] found that polyphenols from *Penthorum chinense* Pursh. can directly bind to Keap1 protein and activate Nrf2 to regulate the expression of downstream antioxidant proteins, thereby reducing vascular inflammation induced by high glucose. Hence, it is our subsequent research work to verify whether polyphenols in GPH extracts can directly interact with the Keap1-Nrf2 signaling pathway by branching docking technique and cell model.

Gut microbiota plays an important role in maintaining the intestinal barrier function, and its imbalance is considered a key factor in the onset of IBD [34,37]. DSS can change the composition of the gut microbiota to aggravate colitis, which is manifested by inhibiting the abundance of probiotics and promoting the growth of harmful bacteria. Intake of dietary active substances, such as polyphenols, is believed to have a positive effect on colitis [12]. Myricetin [14], anthocyanins [26], apple polyphenols extract [17] and honey polyphenols [18] can alleviate DSS-induced colitis in mice by regulating the gut microbiota. The ratio of *Firmicutes*/*Bacteroidetes* (F/B) is an important index of gut microbiota structure change. In this study, DSS treatment significantly reduced F/B values, which was reversed by promoting *Firmicutes* and reducing *Bacteroidetes* after GPH extracts treatment. Enterotoxigenic *Bacteroides fragilis*, which secretes 20 KDa pro-inflammatory zinc-dependent metalloproteinase toxin, is not only related to blood infections and intra-abdominal abscesses, but also a potential instigator of colitis [18,38]. As a probiotic, *Lactobacillaceae* can not only secrete SCFAs, but also activate T-helper to resist foreign pathogens and regulate the secretion of cytokines [31,37]. *Lachnospiraceae* also belongs to SCFA-producing bacteria [21]. Compared with the DDS group, GPH extracts treatment increased the abundance of DSS-inhibited *Lactobacillaceae* and *Lachnospiraceae*, which was related to the increase of SCFAs content. LEfSe analysis showed that *Lactobacillaceae* were one of the key bacteria in the HGPH group. Interestingly, *Akkermansia*, belonging to *Verrucomicrobiaceae*, is controversial bacteria. Y. Zhang et al. [31] believed that *Akkermansia* could stimulate the secretion of pro-inflammatory factors and reduce the production of SCFAs to aggravate colitis due to its mucilage degradation ability; however, Z. Zhang et al. [19] found that chlorogenic acid can improve colitis by promoting the growth of *Akkermansia*. Our results showed that the abundance of *Akkermansia* increased after DSS treatment, which was inhibited by GPH extracts. All changes in the gut microbiota have an impact on the metabolism and information processing of mice.

SCFAs play an important role in maintaining the integrity of the intestinal barrier, providing energy, and regulating intestinal inflammation [31,39]. Disturbance of the gut microbiota caused by colitis can lead to a decrease in the content of SCFAs. GPH extracts treatment reversed the decline in SCFAs levels, especially acetic, propionic, and *n*-butyric acids induced by DSS, which may be related to the recovery of intestinal homeostasis and the increase of SCFAs-producing bacteria, such as *Lactobacillaceae* and *Lachnospiraceae* [31]. SCFAs can exert anti-inflammatory activity through multiple pathways; Pan et al. [40] reported that SCFAs can activate GPRs receptors in epithelial cells to inhibit the expression of pro-inflammatory factors; on the other hand, butyrate can stimulate the PPAR-γ signaling pathway to improve colitis in mice [21]. More interestingly, a recent study showed that SHFAs may act synergistically with the phenolic metabolites in their antioxidant and anti-inflammatory effects [41]. Overall, GPH extracts can maintain gut microbiota homeostasis and alleviate DSS induced colitis by promoting probiotics, inhibiting harmful bacteria, and increasing the content of SCFAs.

## 5. Conclusions

**Figure 6:**
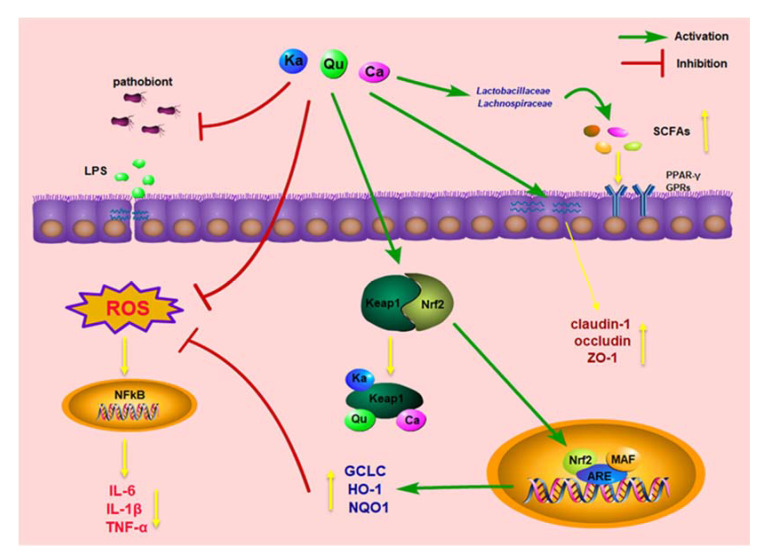
Proposed mechanism and pathway of GPH extracts in ameliorating DSS-induced UC in mice. Qu: quercetin; Ka: kaempferol; Ca: catechin.

In conclusion, the main active substances in GPH extracts were quercetin, kaempferol, catechin and their derivatives. These polyphenols and their metabolites could alleviate DSS induced colitis in mice by activating the Keap1-Nrf2-ARE signaling pathway, regulating gut microbiota, and increasing the level of SCFAs (Figure 6). Our results suggest that polyphenol-rich GPH is a potential dietary resource for the treatment of colitis. On the other hand, GPH contains a lot of dietary fiber, which is also an active substance with positive effects on colitis, and the combined effects by colonic metabolites, i.e., SCFAs and polyphenols/metabolites, will be our focus in the future. Of course, the current research supports GPH as a dietary intervention for UC, and further design of large sample verification and even clinical research are needed.

## Figures and Tables

**Table 1:** Primer sequences.

Gene	Primer	Sequence (5′-3′)	PCR Products
mus GAPDH	Forward	ATGGGTGTGAACCACGAGA	229 bp
	Reverse	CAGGGATGATGTTCTGGGCA	
mus HO-1	Forward	CCTCACTGGCAGGAAATCA	217 bp
	Reverse	TCGGGAAGGTAAAAAAAGC	
mus NQO1	Forward	GGCTGGTTTGAGAGAGTGCT	205 bp
	Reverse	GGAAGCCACAGAAACGCAG	
mus GSTA1	Forward	TCTCAACTACATCGCCACCA	198 bp
	Reverse	TCAAAGGCAGGCAAGTAACG	
Mus GCLC	Forward	AAGCCTCCTCCTCCAAACTC	187 bp
	Reverse	GGGCCACTTTCATGTTCTCG	

**Table 2:** Quantitative results by UHPLC-LTQ-OrbiTrap-MS2 for the major phenolic compounds in GPH extract.

No.	t_R_ (min)	Formula	[M − H]^−^ (*m*/*z*)	Major Fragment Ions (*m*/*z*)	Tentative Identification	μg Quercetin/g GPH Extract
1	3.56	C_25_H_24_O_9_	467.1317	289.1351	(Epi)catechin conjugate 1	496.53 ± 30.60
2	4.25	C_30_H_26_O_14_	609.1241	305.0539, 423.0485, 441.0551, 483.0265, 515.5356, 591.1233	(Epi)gallocatechin dimer	214.68 ± 15.01
3	5.18	C_25_H_24_O_9_	467.1312	289.1352, 305.0813, 351.1628	(Epi)catechin conjugate 2	628.70 ± 40.06
4	6.44	C_20_H_22_O_15_	501.0922	/	Quercetin derivative 1	1409.24 ± 60.01
5	7.43	C_20_H_22_O_15_	501.0922	264.9600, 294.9618, 338.9902, 380.9662, 411.0221, 483.0662	Quercetin derivative 2	1381.57 ± 91.18
6	9.38	C_33_H_40_O_21_	771.1999	609.1423, 285.0310	Kaempferol trihexoside 1	492.17 ± 36.28
7	9.66	C_33_H_40_O_21_	771.2006	609.1426, 285.0313	Kaempferol trihexoside 2	989.83 ± 53.77
8	10.11	C_21_H_20_O_12_	463.0887	301.0334	Quercetin 3-glucoside	45.76 ± 5.02
9	12.64	C_52_H_39_O_25_	1062.7570	1016.7891	Theaflavin derivative	23.34 ± 2.52
10	22.18	C_27_H_32_O_15_	595.2894	222.9419, 240.9657, 279.1984, 315.0365	Isorhamnetin glycoside	294.18 ± 25.20
11	25.71	C_15_H_12_O_5_	271.2285	225.1866	Naringenin	239.15 ± 13.00

## Data Availability

Not applicable.

## Supplementary Materials

The following are available online at https://www.mdpi.com/article/10.3390/foods10112765/s1. Table S1: The criteria of DAI; Table S2: The criteria of histological score.
