# Supplementary material for: Green Pea (Pisum sativum L.) Hull Polyphenol Extracts Ameliorate DSS-Induced Colitis through Keap1/Nrf2 Pathway and Gut Microbiota Modulation
Source: Foods. 2021 Nov 11;10(11):2765. doi: 10.3390/foods10112765 (PMC8624850; doi:10.3390/foods10112765)
Supplement: Supplementary file 1 [file foods-10-02765-s001.zip › foods-1431091-supplementary.pdf]

Supplementary materials

Table S1. The criteria of DAI.

**Body weight loss (%)**	**Stool consistency**	**Rectal bleeding**	**Activity states**	**Score**
No change	Normal	No observable blood	Normal	0
1-5	Loose stool	Small amount of blood in some stool	Some signs of poor condition	1
5-10	Mild diarrhea	Blood in stool regularly seen	Moderately poor condition	2
≥10	Diarrhea	Blood in all stool	Bad condition	3

Table S2. The criteria of histological score.

**Inflammatoy cell infiltration**	**Mucosal injury**	**Crypt distortion**	**Area of lesions (%)**	**Score**
None	None	None	None	0
Mild	Mucous layer	1/3	1-25	1
Moderate	Submucosa	2/3	26-50	2
Severe	Muscularis and serosa	100%	51-75	3
—	—	Entire crypt and surface epithelium destroyed	76-100	4
